# Compassion satisfaction, burnout, and secondary traumatic stress in UK therapists who work with adult trauma clients

**DOI:** 10.3402/ejpt.v4i0.21869

**Published:** 2013-12-30

**Authors:** Ekundayo A. Sodeke-Gregson, Sue Holttum, Jo Billings

**Affiliations:** 1 Redbridge IAPT Service, Ilford, UK; 2 Salomons Centre for Applied Psychology, Canterbury Christ Church University, Tunbridge Wells, UK; 3 Berkshire Traumatic Stress Service, Reading, UK

**Keywords:** Compassion satisfaction, burnout, secondary traumatic stress, Professional Quality of Life Scale, online questionnaire

## Abstract

**Background:**

Therapists who work with trauma clients are impacted both positively and negatively. However, most studies have tended to focus on the negative impact of the work, the quantitative evidence has been inconsistent, and the research has primarily been conducted outside the United Kingdom.

**Objectives:**

This study aimed to assess the prevalence of, and identify predictor variables for, compassion satisfaction, burnout, and secondary traumatic stress in a group of UK therapists (*N*=253) working with adult trauma clients.

**Method:**

An online questionnaire was developed which used The Professional Quality of Life Scale (Version 5) to assess compassion satisfaction, burnout, and secondary traumatic stress and collect demographics and other pertinent information.

**Results:**

Whilst the majority of therapists scored within the average range for compassion satisfaction and burnout, 70% of scores indicated that therapists were at high risk of secondary traumatic stress. Maturity, time spent engaging in research and development activities, a higher perceived supportiveness of management, and supervision predicted higher potential for compassion satisfaction. Youth and a lower perceived supportiveness of management predicted higher risk of burnout. A higher risk of secondary traumatic stress was predicted in therapists engaging in more individual supervision and self-care activities, as well as those who had a personal trauma history.

**Conclusions:**

UK therapists working with trauma clients are at high risk of being negatively impacted by their work, obtaining scores which suggest a risk of developing secondary traumatic stress. Of particular note was that exposure to trauma stories did not significantly predict secondary traumatic stress scores as suggested by theory. However, the negative impact of working with trauma clients was balanced by the potential for a positive outcome from trauma work as a majority indicated an average potential for compassion satisfaction.

It is widely recognised that engaging in trauma work may impact therapists (Figley, 1995, 2002). Whilst some therapists report feelings of well-being from working with trauma clients, the American Psychiatric Association (APA) acknowledges that it is possible to become traumatised indirectly by “learning about unexpected or violent death, serious harm, or threat of death or injury experienced” by another person (2000, p. 463). Two theoretical concepts have been put forward to describe therapists’ experiences: compassion satisfaction (CS) and compassion fatigue (CF).

## Compassion satisfaction

A growing body of literature documents the positive effects of working with trauma. Larsen and Stamm (2008) proposed CS to be “the sense of fulfilment or pleasure that therapists derive from doing their work well” (p. 282). CS is made up of three elements: (1) the level of satisfaction that a person derives from their job; (2) how well a person feels they are doing in their job, related to the levels of competency and control that therapists feel they have over the traumatic material they are exposed to; and (3) the level of positive collegiate support that a person has, with aspects of structural and functional social support being particularly important (Stamm, 2002). Researchers have reported a high potential for CS in mental health professionals in the United States (Conrad & Kellar-Guenther, 2006), in Ireland (Collins & Long, 2003), and amongst interpreters and therapists working at the Treatment Center for Torture Victims in Berlin (Birck, 2001). Additionally, these positive experiences have also been supported by qualitative research (Arnold, Calhoun, Tedeschi, & Cann, 2005; Steed & Downing, 1998).

### Compassion fatigue

The most commonly used terms to describe the negative consequences of working with trauma clients are CF, secondary traumatic stress (STS), vicarious traumatisation (VT), and burnout. A therapist suffering from CF may experience symptoms such as re-experiencing their client's traumatic event, avoidance, or anxiety. CF is believed to develop through prolonged exposure to clients’ traumatic material.

STS is believed to be an acute reaction that develops suddenly, and symptoms are nearly identical to those of clients suffering from posttraumatic stress disorder. VT, however, focuses on the disrupted frame of reference which may permanently impact therapists’ beliefs about others and their “sense of self, world view, spirituality, affect tolerance, interpersonal relationships, and imagery system of memory” (Pearlman, 1999, p. 52). VT is believed to develop through working with several clients over time.

Burnout, unlike the other concepts, is not specifically limited to those working with trauma clients, but is more a reaction to the demands of one's job and environment. It is “a state of physical, emotional, and mental exhaustion caused by long term involvement in emotionally demanding situations” (Pines & Aronson, 1998, p. 9).

Despite some nuances, these concepts are often used interchangeably in the literature. Adams, Boscarino, and Figley (2006) propose that CF is a broad concept which encompasses STS, VT, and burnout as latent clinical features. Alternatively, Stamm (2002, 2009) argues that CF is made up of STS and burnout, so it is evident that some key researchers believe that there are overlaps between these concepts but that CF is believed to be an over-arching concept.

In summary, therapists suffering from CF are hypothesised to experience posttraumatic stress disorder (PTSD) symptoms, disruptions to their cognitive schemas, relational difficulties, as well as physical, emotional, or behavioural distress symptoms. These experiences are believed to affect therapists’ personal and professional relationships and also impact their ability to effectively work with clients (Collins & Long, 2003). To date, research studies provide mixed support for these hypothesised symptoms in therapists working with trauma clients. For example, whilst severe PTSD symptomology has been reported in some therapists (Chrestman, 1999; Kassam-Adams, 1999), milder or subclinical levels have also been observed (Brady, Guy, Poelstra, & Brokaw, 1999; Follette, Polusny, & Milbeck, 1994; Kadambi & Truscott, 2004). Although some studies have documented cognitive disruptions in US therapists (Pearlman & Mac Ian, 1995; Schauben & Frazier, 1995), these have not been replicated by studies in Holland (van Minnen & Keijsers, 2000) and Canada (Kadambi & Truscott, 2004).

### Protective and risk factors

The impact of trauma work appears to vary between individuals, and possible protective and risk factors have been examined. Investigated therapist variables have included age, gender, and engagement in personal therapy, but studies have produced inconsistent findings. For example, whilst some studies have reported that therapists with a personal trauma history experienced higher levels of distress (Kassam-Adams, 1999; Pearlman & Mac Ian, 1995), this finding has not been universally replicated (Schauben & Frazier, 1995).

A mixed picture has also emerged when investigating work-related variables like clinical experience and size of caseload. As exposure to trauma is a prerequisite for CF, one would expect there to be a relationship between these two variables. This has been confirmed by some research which has reported that the percentage of trauma clients on therapists’ caseloads was related to PTSD symptoms (Chrestman, 1999; Kassam-Adams, 1999), burnout, and CF (Craig & Sprang, 2010). Surprisingly, others have not found this hypothesised relationship (Devilly, Wright, Varker, 2009; Meyers & Cornille, 2002; Schauben & Frazier, 1995), and some have found that those seeing more trauma clients reported less distress (Baird & Jenkins, 2003).

Examined organisational factors include the provision of supervision, perceived workplace support, provision of trauma-specific training, urban versus rural workplace setting, remuneration, and working for public versus private organisations. Research findings in these areas are also inconclusive. For example, whilst Pearlman and Mac Ian (1995) reported that therapists not receiving supervision showed more cognitive disruptions, others have found that the amount of supervision received was not related to the experience of traumatic stress or PTSD symptoms (Kassam-Adams, 1999; Meldrum, King, & Spooner, 2002).

Whilst it is possible that these inconsistent findings may be due to the variety of different scales being used to measure therapists’ experiences and the research being carried out on differing self-selecting groups, there is, as yet, no clear picture of the variables which are associated with, or most likely to predict, CF. In fact, a systematic review of the empirical evidence supporting CF, STS, and VT concluded that the quantitative evidence for these concepts was “meagre and inconsistent, relying on small and variable correlations between symptomatic distress and trauma exposure” (Sabin-Farrell & Turpin, 2003, p. 467). Additionally, the majority of research continues to focus on the deleterious effects of trauma work, which has led to a dearth in research on its positive impact and the factors that might promote positive experiences in therapists.

Much of the published research has been carried out in the United States, where, until recently, there was no provision of universal healthcare and where supervision requirements for therapists differ from those in the United Kingdom. With limited research published in the United Kingdom, the question still remains as to whether and to what extent these concepts are contextually valid for UK therapists working with trauma clients.

## Objectives

The objectives for this exploratory study were to:

- investigate the reported levels of CS and CF in a national sample of UK therapists working with trauma clients in specialist trauma and secondary-care services (or similar).
- examine which variables most strongly predict CS and CF.

## Method

### Participants

Therapists working for the UK National Health Service in 50 participating trusts or registered with one or more national professional psychological bodies responded to an online questionnaire between June 2010 and January 2011. All were engaged in trauma work with working-age adults.

Three hundred and forty therapists were recruited. However, 87 questionnaires were excluded from further analysis as therapists either identified themselves as working with children (*n*=4) or with older adults (*n*=1), or dropped out before the end of the questionnaire (*n*=82). Participants were 253 therapists (182 women and 71 men), with 64.5% aged between 30 and 49 years. They either worked in specialist trauma services (22.5%) or secondary-care services (62.5%), or identified themselves as working in “other services” (15%), which included specialist and tertiary services, primary care, private practice, and public or voluntary services. The majority of therapists were clinical or counselling psychologists (69.6%); many had a doctoral qualification (39.1%), over half had worked for less than 10 years (56.2%), and over half identified themselves as having a personal history of trauma (59.3%). The predominant therapeutic approach reported was cognitive-behavioural therapy (CBT) (39.1%), and a large group had had more than a week's trauma-specific training since qualification (47.4%). The majority worked part-time (64%) and had between one and nine trauma clients on their current caseload (65.2%).

### Measures

#### Independent measures

Demographic and background information questionnaire

In addition to demographics, information was sought about therapists’ work setting, core profession, qualifications, caseloads, primary therapeutic modality, trauma training, supervision, perceptions of organisational support, and personal history of trauma.

##### Coping Strategies Inventory

Therapists’ coping strategies were assessed using the two-part Coping Strategies Inventory (CSI; Bober, Regehr, & Zhou, 2006), comprising beliefs and time. The CSI-Beliefs scale explores which coping strategies therapists believe will reduce secondary stress and results in three subscales: leisure, self-care, and supervision which have reported internal reliability coefficients of 0.71–0.82. The CSI-Time scale, which examines the time that therapists spend engaging in activities, results in four subscales: leisure, self-care, supervision, and research and development (R&D), with reported internal reliability coefficients of 0.67–0.80.

#### Dependent measure

##### The Professional Quality of Life Scale, Version 5

The Professional Quality of Life Scale (ProQOL; Stamm, 2009) is a 30-item scale which measures the positive and negative effects experienced by those who choose to help others experiencing suffering and trauma. It is made up of three subscales: CS, CF, and burnout.

The ProQOL asks respondents to rate how frequently they experienced certain feelings in relation to their work with clients in the last 30 days. An example item of CS is “I believe I can make a difference through my work.” An example burnout item is “I feel overwhelmed because my case/workload seems endless,” and a STS item is “I avoid certain activities or situations because they remind me of frightening experiences of the clients I help.” Items are rated on a 6-point scale (which includes 0=*never*, 3=*somewhat*, and 5=*very often*). The alpha reliabilities for the scales have good to excellent reliability (CS α=0.88 [*n*=1,130]; Burnout α=0.75 [*n*=976]; CF α=0.81 [*n*=1,135]).

### Procedure

Ethics approval was gained from the Central London REC 3 Research Ethics Committee. Potential participants were informed about the study by receiving an email from either a Trust representative or their professional body, by e-bulletins from their professional body, on professional body research notice boards, or by being emailed directly.

The email received by all potential participants introduced the study and contained a link to the homepage of the online questionnaire which included additional information about the study and consent information. Once therapists consented to take part in the study by checking the consent box, they were directed to the anonymous online questionnaire which took approximately 10–15 minutes to complete.

### Statistical analyses

A power calculation of required participants was made prior to recruitment. Based on achieving a medium effect size (*R* ^2^=0.13) (as used in Devilly, et al., 2009), with a statistical power of 0.8 (as recommended by Cohen, 1988), and considering the inclusion of up to 12 predictor variables into the planned multiple regressions, we aimed to recruit between 120 and 150 participants. Two-tailed tests were used with a significance value of 0.05. Analyses were conducted using SPSS 17.0 (for Windows 2001).

## Results

### Prevalence of CS, burnout, and STS amongst therapists

Participants’ scores were calculated and categorised into the cut-offs for low, average, and high levels of CS, burnout, and STS in accordance with Stamm's (2009) guidelines (Table 1). Whilst the majority of the therapists scored within the average range for CS and burnout, 70% of the therapists’ scores indicated that they were at high risk of STS, with no therapists scoring low on STS.

**Table 1:** Number of therapists at low, average, and high risk of CS, burnout, and STS (*N*=253)

	Compassion satisfaction	Burnout	Secondary traumatic stress
Low	20 (8%)	25 (9.9%)	0 (0%)
Average	135 (53.2%)	163 (64.2%)	76 (30%)
High	98 (38.8%)	65 (25.8%)	177 (70%)

### Predictors for CS, burnout, and STS

Due to the large number of variables and the exploratory nature of the study, it was decided that only those variables that significantly correlated with CS, burnout, and STS would be entered into the multiple regressions. Therefore, Pearson correlations and point-biserial correlations were performed to identify these variables (Table 2). None of the correlations between the independent variables were above *r*=0.649. CS was negatively correlated with both burnout (*r*=−0.697, *p<*0.001) and STS (*r*=−0.189, *p<*0.003), whilst burnout was positively correlated with STS (*r*=0.454, *p<*0.001).

**Table 2:** Correlations between CS, burnout, and STS and independent predictor variables

	Compassion satisfaction	Burnout	Secondary traumatic stress
Service setting	−0.024	0.008	−0.116
Age	0.265**	−0.200**	−0.043
Gender	−0.039	0.060	0.102
Highest qualification	0.181**	−0.157*	0.051
Number of years post qualification	0.151*	−0.120	−0.135*
Core profession	0.110	−0.095	−0.070
Number of sessions	0.029	−0.035	−0.092
Number of clients on caseload	0.061	0.013	−0.054
Number of trauma-focused clients on caseload	0.119	−0.027	0.120
Predominate therapeutic approach	−0.042	0.027	0.145*
Hours of individual supervision per month	0.031	−0.006	0.187**
Hours of group supervision per month	−0.029	0.006	0.035
Hours of peer supervision per month	0.027	0.034	−0.003
Hours of consultant supervision per month	0.060	−0.039	0.123
Days of trauma-specific training during main training course	0.011	−0.070	0.118
Days of trauma-specific training since qualification	0.201**	−0.155*	−0.054
Personal trauma history	−0.058	0.017	−0.139*
CSI-Beliefs: leisure	0.171**	−0.145*	0.046
CSI-Beliefs: self-care	0.123	−0.099	0.050
CSI-Beliefs: supervision	0.153*	−0.189**	0.013
CSI-Time: leisure	0.048	−0.094	−0.047
CSI-Time: self-care	0.216**	−0.173**	0.172**
CSI-Time: supervision	0.196**	−0.204**	0.115
CSI-Time: R&D	0.282**	−0.192**	0.063
Perceived support by management	0.214**	−0.328**	−0.111
Perceived support by administrative staff	0.102	−0.113	0.063
Perceived support by peers	0.075	−0.155*	−0.057
Perceived support of supervision	0.254**	−0.249**	0.063
ProQOL—compassion satisfaction	1	−0.697**	−0.189**
ProQOL—burnout		1	0.454**
ProQOL—secondary traumatic stress			1

* Note: *p*<0.05;

** *p*<0.01.

Significant correlations shown in bold. CSI=Coping Strategies Inventory; ProQOL=The Professional Quality of Life Scale.

Three simultaneous method multiple regressions were run, one for each of the dependent variables. All variance inflation factors were below 10, tolerance statistics were above 0.2, and casewise diagnostics were reviewed and within accepted parameters as recommended by Field (2009).

#### Predictors for CS

A significant model emerged: *F* (11,220)=5.825, *p<*0.001, explaining 22.6% of the variance (*R* ^2^=0.226) (Table 3). Age, time spent engaging in R&D activities, perceived management support, and perceived supervision support were significant positive predictors of CS. This indicated that older therapists had higher potential for CS. Additionally, the more time that therapists spent in R&D activities (i.e., away from therapeutic work), the higher the potential for CS. As therapists’ perceived level of support from management and supervision increased, so did their potential for CS.

**Table 3:** Regression model for variables predicting CS (*n=*232)

	*B*	*SE B*	*β*	*t*	*p*
Constant	35.19	3.54			
Age	0.80	0.34	0.21	2.39	0.02*
Highest qualification	1.67	1.02	0.11	1.64	0.10
Years of clinical experience	0.38	0.44	−0.07	−0.84	0.40
Trauma training post qualification	0.14	0.25	0.04	0.57	0.58
CSI-Beliefs: leisure	1.51	0.83	0.12	1.82	0.07
CSI-Beliefs: supervision	−1.15	0.89	−0.10	−1.30	0.19
CSI-Time: self-care	1.06	0.73	0.11	1.46	0.15
CSI-Time: supervision	−2.9	1.17	−0.02	−0.25	0.80
CSI-Time: R&D	1.81	8.2	0.17	2.21	0.03*
Perceived management support	0.84	0.38	0.14	2.22	0.03*
Perceived supervision support	1.31	0.56	0.17	2.34	0.02*

*R* ^2^=0.226 (*p*<0.001).

* *p*<0.05.

Adjusted *R*^2^=0.187. CSI=Coping Strategies Inventory.

#### Predictors for burnout

A significant model emerged: *F* (10,226)=7.243, *p<*0.001, which explained 24.3% of the variance (*R* ^2^=0.244) (Table 4). Perceived management support and age were significant negative predictors of burnout. Being older appeared to be a protective factor against burnout. Additionally, as therapists’ perceptions of management support increased, this was related to a decreased risk of burnout.

**Table 4:** Regression model for variables predicting burnout (*n=*237)

	*B*	*SE B*	*β*	*t*	*p*
Constant	69.59	3.31			
Age	−0.53	0.26	−0.15	−2.05	0.04*
Highest qualification	−1.21	0.94	−0.08	−1.29	0.20
Trauma training post qualification	−0.09	0.23	−0.03	−0.39	0.70
CSI-Beliefs: leisure	−0.81	0.77	−0.07	−1.05	0.30
CSI-Beliefs: supervision	0.82	0.83	0.07	0.99	0.32
CSI-Time: self-care	−0.91	0.67	−0.10	−1.36	0.18
CSI-Time: supervision	−0.53	1.07	−0.04	−0.49	0.62
CSI-Time: R&D	−0.53	0.75	−0.05	−0.71	0.48
Perceived management support	−1.59	0.36	−0.29	−4.39	0.001**
Perceived peer support	−0.26	0.51	−0.04	−0.52	0.61
Perceived supervision support	−1.00	0.54	−0.14	−1.86	0.06

*R*^2^=0.244 (*p*<0.001).

* *p*<0.05;

** *p*<0.001.

Adjusted *R*^2^=0.206. CSI=Coping Strategies Inventory.

#### Predictors for STS

A significant model emerged: *F* (5,239)=5.286, *p<*0.001 which accounted for 10.0% of the variance (*R* ^2^=0.100) (Table 5). Time spent in individual supervision and time spent engaged in self-care were significant positive predictors for STS. Therefore, those therapists who spent more time both in supervision and in self-care activities were at higher risk of STS. Additionally, those therapists that had experienced a traumatic event themselves were at higher risk of STS.

**Table 5:** Regression model for variables predicting STS (*n*=245)

	*B*	*SE B*	*β*	*t*	*P*
Constant	60.01	2.67			
Years of clinical experience	−0.63	0.36	−0.12	−1.75	0.08
Therapeutic model	1.55	0.92	0.10	1.68	0.10
Time spent in individual supervision	1.53	0.75	0.14	2.06	0.04*
Personal trauma history	−2.04	0.96	−0.14	−2.12	0.04*
CSI-Time: self-care	1.22	0.60	0.13	2.03	0.04*

*R*^2^=0.100 (*p*<0.001).

* *p*<0.05.

Adjusted *R*^2^=0.081. CSI=Coping Strategies Inventory.

## Discussion

This exploratory study aimed to investigate indicators of the prevalence of both positive and negative experiences associated with working with trauma clients for therapists in the United Kingdom. Whilst the majority of therapists reported average potential for CS and average risk of burnout, 70% of therapists had scores that suggested they were at high risk of STS. Higher risks of burnout were associated with higher risks of STS, and they were both associated with a lower potential for CS. Maturity, time spent engaging in R&D activities, and a higher perceived supportiveness of management and their supervision predicted higher potential for CS in therapists. Conversely, youth and a low perceived supportiveness of management were risk factors for burnout. Therapists who spent more time engaged in individual supervision and self-care activities, and who had a personal history of trauma, reported higher risks of STS.

Past studies have used a range of different outcome measures to study these concepts, making direct comparison of prevalence rates difficult. However, the ProQOL-III (an earlier version of the outcome measure used in the present study) was used by Craig and Sprang (2010) with self-identified trauma specialists in the United States. Whereas they found that only 5% of their 508 therapists were at high risk of burnout and STS, the present study's findings were drastically different, with 25.8% at high risk of burnout and 70% at high risk of STS. For CS, 53.2% of therapists in the present study had an average potential for CS, whilst 38.8% had high potential for CS. Whilst lower than Craig and Sprang's (2010) study, which reported that 46% of their therapists scored high in CS, this is encouraging as it indicates that a large number of therapists enjoy their work with trauma clients and adds to the growing research evidence suggesting there may be a positive impact of trauma work for therapists.

The proportion of therapists at high risk of burnout and STS was much higher in this study than in other studies utilising different measures (Birck, 2001; Kassam-Adams, 1999; Meldrum et al., 2002; Meyers & Cornille, 2002; Wee & Myers, 2002). The reason for the higher level of burnout and STS in UK therapists is not immediately apparent. Participants in the present study were similar to those in Craig and Sprang's (2010) sample, although other authors have surveyed mental health professionals who may not actively engage in therapy with clients. Craig and Sprang (2010) surveyed those who identified themselves as having some expertise in trauma treatment, 44.3% of their sample being psychologists and 46.3% from a social work background. In the present study, only 2% of the sample came from a social work background, with the majority (69.6%) being either clinical or counselling psychologists. More research is needed to ascertain what impact different professional training may have on levels of CS, burnout, and STS.

As with Craig and Sprang (2010), the present study found youth to be a risk factor for burnout. In addition, older therapists reported higher potential for CS. Therapists in the present study were younger than those of Craig and Sprang (2010), so this may account for the lower levels of CS and higher levels of burnout reported in this study. Other researchers have found that years of clinical experience predicted higher potential for CS (Craig & Sprang, 2010), lower levels of cognitive disruptions (Pearlman & Mac Ian, 1995), lower levels of avoidance, dissociation, anxiety (Chrestman, 1999), and higher levels of emotional exhaustion (Baird & Jenkins, 2003). As clinical experience did not significantly predict CS, burnout, or STS in the present research, it appears that maturity and life experience, as opposed to clinical experience per se, were more important in predicting CS and burnout in our sample. It may be that these older clinicians have remained in the field as they have found a way of coping with the demands of trauma work.

Interestingly, perceived supportiveness of supervision as opposed to the provision of supervision predicted a higher potential for CS which may suggest that the quality of supervision was more important than the quantity. In stark contrast with previous research, the more individual supervision therapists were receiving, the higher their risk of STS. It may be that those therapists who were in distress actively sought out more supervision than those who were coping better, or indeed their managers may have insisted on more supervision for the therapists they perceived as not coping. Although the use of supervision has been recommended to ameliorate the negative effects (Pearlman & Mac Ian, 1995; Pearlman & Saakvitne, 1995; Sexton, 1999), less work has been done to identify the elements that constitute good supervision for trauma work. Through her supervision of those who work with sexual abuse clients, Etherington (2009) suggests that supervision should focus on “the interrelationship between the trauma itself, the person of the counsellor, the helping relationship … and the context in which the work is offered” (p. 183). This seems a good start point; however, more research is needed to further clarify what elements are integral to the supervision of trauma therapists.

The literature suggests that engaging in self-care activities lowers the risk of STS (Rothschild, 2006). Therefore, the finding that those who spent more time engaging in self-care activities were at a higher risk of STS appears surprising. Whilst it is possible that therapists who were struggling more actively engaged in self-care activities in an attempt to alleviate their distress, another alternative explanation may be that these activities had indeed reduced therapists’ risk of STS but that the present study was unable to capture this due to the cross-sectional design of the study. Further research is needed to explore these findings.

Higher perceived levels of management support predicted lower risk of burnout and higher potential for CS. These findings support the theoretical underpinnings of CS (which propose positive collegiate support as an integral part of CS) and burnout (where personal accomplishments are linked to lack of resources such as poor social support) (Schaufeli & Enzmann, 1998). Previous research has also found that therapists who rated the emotional and technical support afforded to them at work as high, exhibited lower scores of work stress (Kassam-Adams, 1999). Although management support was clearly an important part of therapists’ functioning, the present research did not tease out the aspects of management support that were deemed most helpful. It is also interesting that perceived management support appeared to predict CS and burnout which are not exclusive to trauma therapists, suggesting that perceived management support may be important to therapists in general as opposed to trauma therapists per se. Future research should explore the different elements that make up perceived management support and investigate the different ways in which this can be cultivated in services.

As a coping strategy, time spent in R&D activities was found to positively predict the potential for CS. Perhaps this time spent away from direct client work helps therapists bring a balance to their work life. Indeed, Chrestman (1999) found that those therapists who spent more time doing clinical work as opposed to other activities reported increased avoidance, whilst those who spent more time in research activities reported decreased avoidance.

Mental health professionals report a higher prevalence of personal trauma than other professionals (Follette et al., 1994). Almost 60% of participants reported having a personal trauma history and were shown to be at higher risk of STS. These findings were consistent with research by Pearlman and Mac Ian (1995) and Kassam-Adams (1999) which suggests that therapists’ previous experience of trauma may contribute to making them more vulnerable to the trauma stories of others. However, it should be highlighted that some research has contradicted these findings (e.g., Schauben & Frazier, 1995). Regardless, future research is needed to ascertain whether therapists with a personal trauma history require any additional support in their work and indeed what support would be most effective.

It is notable that the number of trauma clients on therapists’ caseloads did not predict STS as would be predicted by the theories of CF, STS, and VT and which has been reported by researchers (Chrestman, 1999; Kassam-Adams, 1999). Other researchers have also not found this theorised relationship (Devilly et al., 2009; Meyers & Cornille, 2002). This calls into question whether it is indeed the exposure to clients’ trauma stories that causes therapists’ distress, whether it is therapeutic work in general, or if other factors are the cause. As van Minnen and Keijsers (2000) have found STS symptoms in therapists not working with trauma clients, they have argued that “the negative effects of trauma work, reported in previous studies, may have been overestimated” (p. 197).

The National Institute for Clinical Excellence (NICE) guidelines for PTSD (2005) recommend the use of trauma-focused CBT and eye movement desensitisation and reprocessing (EMDR) for adult trauma clients. In this study, 39.1% of participants worked with trauma clients using CBT and 12.6% used EMDR, whilst the rest used other models. Increased CS and reduced burnout and STS have been found to be associated with evidence-based practice (Craig & Sprang, 2010). Therefore, the finding that almost 50% of participants were making use of non-NICE-evidenced models may also be contributing to the higher risk of burnout and STS seen in this study. Additionally, therapists with special trauma training have been found to report significantly more CS and less burnout than those who did not have training (Craig & Sprang, 2010). Although a large group had over a week's specific trauma training, future research should focus on therapists’ competence to deliver trauma therapy and the impact that this may have on their risk of burnout and STS as well as their potential for CS.

### Contextual issues

The majority of therapists (86%) in this study worked within the UK National Health Service (NHS), and the structure of psychological provision in the United Kingdom differs from that around the world. It is likely that this may have impacted therapists’ experiences of their work. The present study was not able to ascertain the contribution that the NHS structure may have had on the reporting of these concepts. Further research should investigate the differences between reported levels of CS, burnout, and STS in therapists working for the NHS and the private or voluntary sector and explore whether different predictors exist in these varied work settings.

It is also important to remember the context of the present study. Data were collected during a challenging period for the NHS when the United Kingdom was in recession and the newly formed coalition government had announced changes to the NHS which included “up to £20 billion of efficiency savings by 2014,” the reduction of “NHS management costs by more than 45% over the next four years,” and the restructuring of commissioning with abolishment of Primary Care Trusts to be replaced by GP (general practitioner) consortia (Department of Health, 2010, p. 5). Anecdotally, this meant that many NHS posts were being cut, under threat, or being restructured, and less money was available for training. It is, therefore, possible that these extraneous factors may have affected these results, in particular burnout which is more related to general work stressors.

### Methodological issues

The recruitment method chosen made it impossible to ascertain a response rate, and it was not possible to know if those who chose to participate in the study differed from those who chose not to. The findings of this study are therefore made with caution as it is not known how representative this sample is of UK therapists working with trauma clients.

Although the online methodology has been used to assess trauma and PTSD symptoms in the general population (e.g., Butler et al., 2005), this is one of the first studies to use this methodology to assess levels of CS, burnout, and STS in therapists. 52.3% found the online methodology *not difficult/easy* to complete, and 17.3% found it *extremely easy*. Additionally, the overwhelming majority of therapists (84.9%) reported being either *quite comfortable* or *extremely comfortable* in answering these types of questions online. However, almost a quarter (24%) of those who started the questionnaire dropped out before the end, making their data unusable, and a third of those dropped out immediately after consenting to take part in the questionnaire. Future research should include the provision for participants to save their responses and complete the questionnaire at a later date as this may improve dropout rates.

This study attempted to access those most likely to work with trauma clients by targeting adult specialist trauma services, secondary-care services, as well as those who identified themselves as having a special interest in trauma from professional bodies. It is acknowledged that this sampling procedure excluded a large group of therapists working in child and adolescent teams, older adult services, other specialist services, and primary care. Additionally, the lack of a control group means that it is not possible to ascertain whether these findings are indeed limited to those working with trauma clients. Future research should, therefore, not only consider comparative studies between therapists working with these different client groups as research suggests differences may be present (Dyregov & Mitchell, 1992; Figley, 1995; Hopkins, 1998) but also compare trauma therapists with therapists working with different client presentations.

### Implications for clinical settings

Therapists working with trauma clients should be made aware of the possibility of being negatively impacted from working with this client group. Specific risk factors identified in the present research that therapists may want to be aware of include having a personal trauma history and being younger in age. However, they should also be informed that there is the potential for CS and personal growth.

As with all therapeutic work, it is important for therapists to monitor their own well-being and be mindful of the well-being of their colleagues, as being negatively impacted can affect their therapeutic work and professional and personal relationships. Therapists should remain aware of their own possible triggers. It may be helpful for therapists to consider triggers in different domains, including the personal, professional, and environmental domains (Yassen, 1995).

Perceived management support was found to be a positive predictor of CS and a negative predictor of burnout. Therefore, management appears to have a role to play in therapists’ well-being. This study was not able to identify the specific elements that constitute good management support, and this is an area for future research. It is therefore advisable for managers to consult with the therapists in their service who work with trauma clients to determine what support the therapists feel they require from management. Areas that management should consider include organisational culture, workload, work environment, trauma-specific education and training, group support, supervision, resources for self-care (Bell, Kulkarni, & Dalton, 2003), and workplace context. For example, Chrestman (1999) found that lower levels of vicarious trauma were associated with a more diverse caseload.

Supervision is an area that the present research suggests may be advisable for management to review. Services should consider auditing their supervision provision as these results suggest that the perceived supportiveness of supervision, which may be related to the quality of supervision, may be more important to therapists’ well-being, specifically therapists’ potential for CS, than the quantity of supervision.

## Conclusions

This study made a first attempt at exploring the prevalence of CS, burnout, and STS in a sample of UK-wide therapists who worked with adult trauma clients. The findings paint a different picture from those reported by international colleagues, with a large number of therapists in the United Kingdom seemingly at high risk of STS. However, the majority of therapists’ scores suggested average potential for CS and risk of burnout. The study highlighted factors which predicted levels of CS, burnout, and STS in therapists. Of particular note was that exposure to clients’ trauma stories, measured by the number of trauma-focused clients in therapists’ caseload, was not found to be a predictor of STS, as has been hypothesised, suggesting that the distress experienced by therapists in this sample may have been due to other factors. Whilst there were contextual and methodological limitations to this research, there is a need for further research to explore and replicate these findings in a representative sample of UK therapists. Attention also needs to be given to finding ways to support therapists who may be in distress.

This was the first large-scale study in the United Kingdom, and the first to use an online questionnaire, to explore the positive and negative experiences of therapists working with trauma clients. Further research is needed to continue building our knowledge in this area.
